# Reproducibility of Glucose Fluctuations Induced by Moderate Intensity Cycling Exercise in Persons with Type 1 Diabetes

**DOI:** 10.1155/2021/6640600

**Published:** 2021-03-30

**Authors:** Gabriel Tafdrup Notkin, Peter Lommer Kristensen, Ulrik Pedersen-Bjergaard, Andreas Kryger Jensen, Stig Molsted

**Affiliations:** ^1^Department of Clinical Research, Nordsjællands Hospital, Denmark; ^2^Department of Endocrinology and Nephrology, Nordsjællands Hospital, Denmark; ^3^Department of Clinical Medicine, Faculty of Health and Medical Sciences, University of Copenhagen, Copenhagen, Denmark; ^4^Section of Biostatistics, Department of Public Health, University of Copenhagen, Denmark

## Abstract

**Aims:**

The purpose was to assess the reproducibility of glucose changes during three sessions of standardized moderate intensity continuous training of cycling on an individual level in people with type 1 diabetes.

**Methods:**

Twelve adults (six females) with type 1 diabetes performed three test sessions on an ergometer bicycle (30 min, 67% of predicted heart rate) on three different days. The participants were 36.5 (26.6-45.5) (median, IQR) years old, and their HbA1c was 65 ± 15 mmol/mol (mean ± SD). Two hours before the tests, the participants had a standard meal. Interstitial glucose (IG) and capillary glucose (CG) were measured using an iPro2 Medtronic continuous glucose monitor and the Bayer Contour XT-device, respectively. Prior to the test sessions, resting heart rate was measured using a digital blood pressure monitor to estimate the desired intensity of the exercise.

**Results:**

The average within-participant relationship between the average slope in glucose during sessions 2 and 1 was in IG -0.29 (95% CI -1.11; 0.58) and in CG -0.04 (-0.68; 0.77). Between sessions 3 and 2, IG is 0.18 (-0.27; 0.64) and in CG 0.13 (-0.25; 0.55). Between sessions 3 and 1, IG was 0.06 (-0.57; 0.71) and in CG 0.06 (-0.39; 0.52). The results indicate low reproducibility at participant levels and remained unchanged after adjustment for baseline glucose values.

**Conclusion:**

On an individual level, the glucose declines during three standardized sessions of PA were not associated with identical responses of the measured IG and CG levels. An overall anticipated decline of glucose concentrations was found in the moderate intensity cycling sessions. This highlights the importance of regular CG measurements during and after physical activity and awareness towards potential exercise-induced hypoglycemia in persons with type 1 diabetes.

## 1. Introduction

Physical activity (PA) is recommended by the American Diabetes Association as part of the diabetes treatment [1]. Previous studies have reported positive effects of PA on insulin requirements, the lipid profile, physical fitness, muscle strength, and the risk of developing cardiovascular disease in people with type 1 diabetes mellitus (T1DM) [2]. Exercise is however associated with glycemic fluctuations including hypoglycemia and hyperglycemia in T1DM [3–10]. Indeed, hypoglycemia in relation to exercise may be a challenge for the person with T1DM who wishes to maintain or increase the level of PA and may be a barrier to exercise training in T1DM [11].

Plasma glucose (PG) fluctuations in T1DM are a result of several factors, among them food intake [12], rate of endogenous production and uptake of glucose, insulin sensitivity, and insulin timing, type, and dose. In addition, there may be biological variations between individuals. Given the variety of factors affecting PG, the day-to-day variation in glucose responses to exercise could be significant. Previous studies have investigated the reproducibility of blood glucose (BG) or PG patterns during different exercise types and intensities and have reported inconsistent results between exercise sessions in people with T1DM [13–15]. These previous studies were limited by the use of two exercise sessions and by inclusion of primarily adolescents [13, 14]. In the study of adults by Biankin et al., the PG was measured with an interval of 15 minutes during a 45-minute exercise session and found to be reproducible in two exercise sessions in a fasted state (defined as 12 h fast, with the previous evening's intermediate-acting insulin reduced by 2 units and without injection of morning insulin) [15]. Continuous glucose monitoring (CGM) has made it possible to monitor interstitial tissue glucose levels with high time resolution and accuracy without the need of multiple capillary glucose (CG) measurements conducted by the individual. With five minutes intervals, the CGM systems estimate the interstitial glucose (IG) level, and it has been suggested that CGM systems are valid technologies to monitor glucose values during exercise [10, 16, 17], although recent studies have shown certain delays in the measurements as well as inaccuracy during high intensity interval training [18–20].

While CGM may provide comprehensive data on the IG changes induced by exercise training, the reproducibility of these changes determined by CGM data is not clear. Thus, the aim of this study was to investigate the reproducibility of the interstitial tissue glucose response using CGM with CG for calibration during three sessions of moderate intensity continuous training of cycling in adults with T1DM. The hypothesis was that three standardized test sessions on three different days would stimulate identical glucose changes during PA assessed by CGM and CG measurements in persons with T1DM.

## 2. Materials and Methods

This mechanistic, observational study was performed in The Clinical Research Unit, Nordsjællands Hospital, Denmark. All tests were conducted by the same research physiotherapist (GTN). The study was performed within five months, and the participants were tested from mid-December 2016 till May 2017. The standardized testing and measurement procedures were used in accordance with the study protocol NCT02942069 available on ClinicalTrials.gov. Permission to carry out the study was granted from the Local Committee on Health Research Ethics (H-16043041). The study was conducted in accordance with the Helsinki Declaration [21, 22]. The protocol was approved by the Data Protection Agency.

### 2.1. Subjects

People with T1DM from the diabetes outpatient clinic at Nordsjællands Hospital (service for around 1000 patients) treated with or without an insulin pump were invited to participate in the study while they were in the clinic for an appointment. Recruitment through Facebook groups was also used. No incentives were offered for participation. The inclusion criteria were T1DM for > two years, 18-60 years of age, and regular exercise of at least 30 minutes duration, during which the participants would feel exhausted, one or more times a week. The participants should agree to follow the study protocol with regard to the standardized exercise and to use CGM and measure CG during the study. Exclusion criteria were pregnancy, alcohol or drug abuse, treatment with glucocorticoids or beta-blockers, or regular exercise at a high level (five or more times a week with high intensity).

The study included three visits: at visit 1, the participants were informed and trained in the insertion and use of iPro2 Medtronic CGM equipment and introduced to the first test session. Participants were instructed in inserting the CGM preferably in the subcutaneous region of the abdominal area at least 7.5 cm from their insulin-injection sites [23]. They were also instructed to measure CG with a Bayer Contour XT-device four times daily prior to the three regular meals and before going to sleep to calibrate the CGM equipment. The participants mounted the CGM equipment by themselves before the second and third visit two hours before the test session. The CGM data were blinded to the participants. All CGM and CG data were recorded and registered in the iPro 2 Medtronic system and uploaded in an online database (Carelink) provided by Medtronic. The participants reported in a diary how physically active they were prior to the first test session. The data were used to estimate the amount of recreational physical activity during the week as part of the description of participants' characteristics. The standardized meal and physical activity during leisure time were self-reported, and attempts were made to have this recorded in written logbooks throughout the study period.

Visits 1-3 comprised three identical test sessions with 30 min PA on a Monark Ergometer bicycle and took place in the clinical research facilities at Nordsjællands Hospital. On the day before the tests, the participants were not allowed to do any exercise training or to consume alcohol. Two hours before the test sessions, they had a self-chosen standardized (in regard to content and amount) breakfast meal or lunch and an insulin dose that were identical at the three sessions. Before the first session, resting heart rate and blood pressure were measured using a Microlife BP A3 Plus digital blood pressure monitor to estimate the desired intensity of the PA in the three sessions. The maximum heart rates of the participants were calculated using the following formula: 208 − (age∗0.7) [24]. The heart rate reserve (difference between the maximum and resting heart rate) was calculated, and the difference was multiplied by 0.7 and added to the resting heart rate to calculate the target heart rate during the tests [25]. The target heart rate was determined to be the same at the three test sessions.

The tests consisted of five minutes of warm-up on the ergometer followed by 30 minutes of PA at ~70% of the participant's estimated work capacity. The testing took place on scheduled days with one week between each test session. The test times were preferable before noon and at the same time of the day for the three test sessions. As some participants were not able to be tested before noon, they were tested after noon. The participant's heart rate was monitored constantly using a Polar FT1 Heart Rate Monitor and chest strap, and participants were continuously instructed to keep it at the desired target level throughout the 30 min session. The participants worked with a cadence of approximately 70 revolutions per minute (RPM), and this was identical in the individual's three tests. The overall output power in watt performed on the ergometer bicycle was registered during the test sessions. The CG was measured with the Bayer Contour XT-device that the participants received from the beginning of the tests (prior to warm-up on the ergometer bicycle) and after every five min throughout the 30 min of continuous training of cycling.

### 2.2. Statistics

The change in CGM-based glucose values, that is interstitial glucose (IG), was primary endpoint. Observed glucose trajectories were analyzed using multivariate linear mixed effects regression for all participants and the three tests jointly, but separate models were fitted for the IG and CG glucose trajectories. A quadratic polynomial relationship between glucose and time was fitted at both the population and participant level. The participant-specific random effects were assumed to follow a zero-mean multivariate normal distribution with an unstructured 9 × 9 covariance matrix.

Baseline values for each session were estimated by the zero-order coefficient in the parameterization of fixed and random trajectories. Estimated average slopes for each participant and test were calculated as the average derivatives across the test duration and given by linear transformations of fixed and random effects.

Agreement between the participant-specific slopes across tests was assessed by the regression line of the random average slopes from one test against another and was estimated from the covariance matrix of the random effects by the properties of the multivariate normal distribution. The associations between the average slopes were also adjusted for the individual baseline values. The software used for the statistical analyses were STATA 15 [26], R [27], and Stan [28].

## 3. Results

All 12 participants completed all the test sessions. Characteristics of the participants are presented in Table 1. Nine participants used insulin pump, and six of the participants were female. It was possible to replicate the exercise stimulus in sessions 1, 2, and 3 for all participants.

### 3.1. Glucose Changes during Exercise

**Figure 1:**
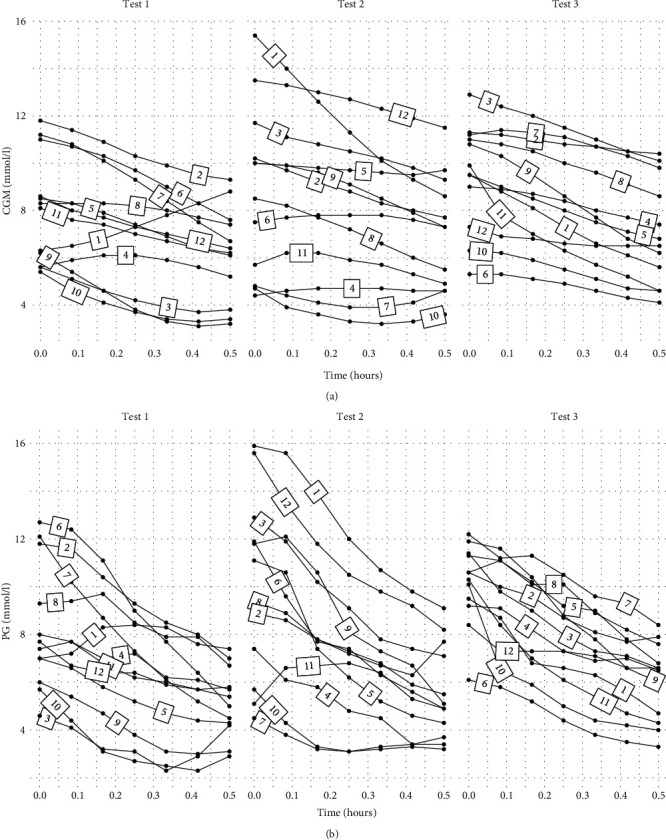
(a) Interstitial glucose levels measured by continuous glucose monitoring during the three 30 min exercise tests (tests 1, 2, and 3) in 12 people with type 1 diabetes. The numbers on each line defines the identification-number of the participant. (b) Capillary glucose levels during the three 30 min exercise tests (tests 1, 2, and 3) in 12 people with type 1 diabetes. The numbers on each line defines the identification-number of the participant.

The participants' IG decreased from 8.0 ± 2.3 to 6.2 ± 2.0 mmol/l, *p* = 0.003, in test 1; from 8.9 ± 3.6 to 7.0 ± 2.4 mmol/l, *p* = 0.007, in test 2; and from 10.1 ± 1.7 to 9.5 ± 2.2 mmol/l, *p* < 0.001, from test 3. There was no difference between the glucose decline in the three test sessions on a group level. In Figure 1(a), all participants' IG responses during the three test sessions are presented. In 35 of the 36 experiments, IG declined during cycling. In one experiment, which was the very first of all sessions, a great IG increment was observed. As we consider unaccounted bias to be the reason for this adverse response, we have omitted this particular test from the following CGM analyses. In Figure 1(b), the participants' CG responses are presented.

The estimated populations' means of baseline glucose and the average slope of IG and CG during the three test sessions are presented in Table 2 along with the between-participant standard deviations.

### 3.2. Comparison of Glucose Changes during Exercise at the Participant Level

**Figure 2:**
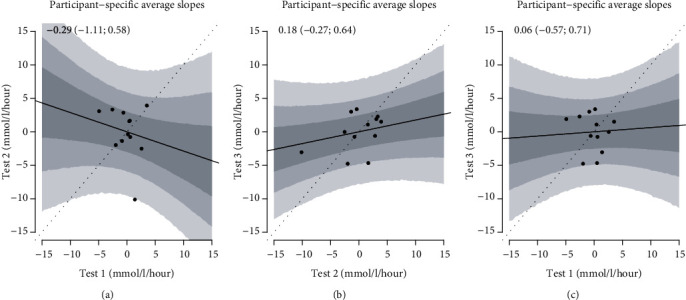
The figure shows the participant-specific continuous glucose monitor average slopes in tests 1 and 2 (a), tests 2 and 3 (b), and tests 1 and test 3 (c). The regression line is the relationship between average slopes across trials, and the gray regions are 50%, 80%, and 95% confidence intervals. If the tests had elicited identical glucose changes, all points had been on the dashed identity line. The values at the top are the slopes of the regression lines with 95% confidence intervals.

**Figure 3:**
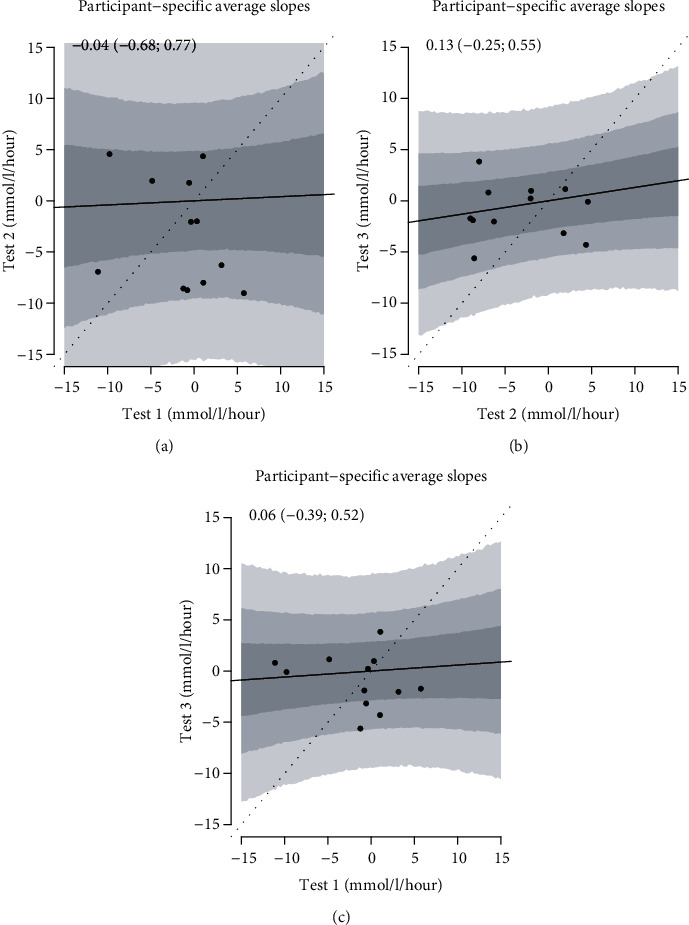
The figure shows the participant-specific capillary glucose average slopes in tests 1 and 2 (a), tests 2 and 3 (b), and test 1 and test 3 (c). The regression line is the relationship between average slopes across trials, and the gray regions are 50%, 80%, and 95% confidence intervals. If the tests had elicited identical glucose changes, all points had been on the dashed identity line. The values at the top are the slopes of the regression lines with 95% confidence intervals.

The estimated participant-specific average slopes in IG from session 2 plotted against the corresponding values from session 1 are presented in Figure 2(a) and similarly in Figure 2(b) for session 3 against session 2. In Figure 2(c), session 3 is plotted against session 1. The participant-specific average slopes of the CG measurements are presented in Figures 3(a)–3(c). If the average slopes were in perfect agreement across the three tests, they would follow close to the identity line, hence a linear relationship.

As it can be seen from the figures, the relation between the slopes of glucose during the PA sessions was poor within participants, indicating that the glucose changes in the three sessions were not alike although the PA stimuli were. As baseline glucose levels may independently affect the glucose changes during PA, we adjusted for baseline glucose, and this did not change the results. The linear relationship between participant-specific average slopes in IG at test 2 against test 1 was -0.29 (-1.11; 0.58); at test 3 against test 2, it was 0.18 (-0.27; 0.64), and at test 3 against test 1, it was 0.06 (-0.57; 0.71). For CG, they were -0.04 (-0.68; 0.77) for test 2 against test 1, 0.13 (-0.25; 0.55) for test 3 against test 2, and 0.06 (-0.39; 0.52) for test 3 against test 1.

Values at 3.9 mmol/l or below due to hypoglycemia may induce a hormonal counter regulation [29] with increased glucose levels and affected slopes. However, when values at 3.9 mmol/l or below were removed in the analyses, the results remained unchanged (data not shown). No test sessions were cancelled due to hypoglycemia.

## 4. Discussion

This study shows pronounced day-to-day variability in the CGM-recorded glucose response to a standardized 30 min PA session at 67% of the predicted max heart rate on a stationary ergometer in people with T1DM. Adjusting for glucose levels immediately before physical activity did not change the results.

The remarkable variability in glucose response was observed even though several factors were attempted standardized in this study, e.g., carbohydrate intake before PA, morning insulin dose, no exercise the day before the standardized PA sessions, and timing and amount and intensity of PA. Although some of these factors may also be standardized to a certain degree in real life PA, very often some of the factors are not. This could indicate that the glucose levels during PA in real life could be even more unpredictable than in the present study. The result is important from a clinical point of view: It stresses the importance of easy access to CG measurements during PA, as the person with T1DM can probably not foresee the glucose trajectory with the desired precision from training session to training session.

The effect of PA on CG in persons with T1DM is associated with several factors. First, the CG response during a PA session is affected by the type and dose of insulin, food intake, and PA before the session. In this study, a meal and a bolus of insulin were taken two hours before the experiment, which means that the responses may be influenced by carbohydrate absorption and action of the bolus insulin. The participants were asked prior to each test session with regard to their meal in order to standardize this throughout the three test sessions. The fed situation has been suggested to reduce the reproducibility of CG tests during exercise training in persons with T1DM [15]. In addition, the response is however also affected by other hormones than externally provided insulin. Sleep quality, stress, illness, competition, and exercise timing on the day may affect hormones including growth hormone, glucagon, adrenalin, and cortisol, which then can affect blood glucose [30–34].

It is important to note that the result of this study was achieved using aerobic sessions of PA with a moderate and constant intensity. Thus, it is not clear whether the results are reproducible if they were tested during strength training or high intensity interval training sessions. Strength training and high intensity interval training have the potential to lead to an increase in blood glucose, which is the opposite response than shown in the present study with moderate intensity continuous training of cycling [35].

The results of this study should be interpreted with caution due to the relatively low number of participants. Furthermore, the results were achieved in a sample selected with specific criteria, among them a maximum age of 60 years and an exclusion of persons with a relatively high level of PA. Moreover, the characteristics of the sample may differ from the general population with T1DM as nine of 12 participants used insulin pumps, compared to the 13% of the T1DM population in the US [36]. In Denmark, the fraction of patients with T1DM who are treated with an insulin pump is not known; however, it is probably less than 50% based on experience from our own clinic. Finally, CGM sensors were mounted relatively close to the moderate intensity continuous training of cycling, which may have reduced precisions of the sensor. However, this could also reflect real-life situations for persons with T1DM as they would not wait 24 hours after mounting a new CGM sensor before performing PA.

The inclusion of persons with self-reported moderate levels of PA may be a strength in the study as the results may be representative to a broad sample of persons with T1DM. However, the participants' level of PA per week showed considerable variation and could explain the variation in power output on the ergometer bicycle during the test sessions. In addition, the study results were based on three PA sessions instead of two sessions, which were used in previous studies of the reproducibility of changes in CG in relation to exercise in T1DM [13–15].

In almost all test sessions, there was a decline in the IG as anticipated, a result that should be included in the counseling to the patient in clinical practice. Our results imply that identification of exercise- and person-related factors which induce glucose changes is important. This could help us build algorithms or easy-to-use rules of thumb for instant patient-directed advice on intake of carbohydrates and insulin doses before, during, and after exercise.

In conclusion, at an individual level, the response of interstitial and capillary glucose levels to three identical PA sessions with standardized work intensity, food intake, and insulin use on the day of the sessions was variable. Thus, persons with T1DM must rely on glucose measurements or CGM during exercise training to avoid unwanted glycemic excursions. An overall anticipated decline of glucose concentrations was found in the moderate intensity cycling sessions. Awareness regarding exercise-induced hypoglycemia should therefore be highlighted to participants with T1DM.

## Figures and Tables

**Table 1:** Characteristics for 12 participants with type 1 diabetes.

Variable	*N* = 12
Women (*n*, %)	6 (50%)
Age (years)	36.5 (26.5-45.5)
BMI (kg/m^2^)	27.6 ± 4.7
Duration of diabetes (years)	16 ± 9.3
Insulin pump (*n*, %)	9 (75%)
HbA1c (mmol/mol)	65 ± 15
HbA1c (%)	8.1 ± 3.5
Basal-insulin dose (units/per day)	22 ± 7.4
Physical activity per week (min/week)	167 ± 82.5
Average power output during exercise (watt)	141 ± 47.6
Average percent of max heart rate^∗^ (%)	67 ± 7.5

^∗^Percent of max heart rate during the tests. Data are presented as mean ± SD and or median (interquartile range).

**Table 2:** Estimated population means and between-participant standard deviations for baseline continuous glucose monitoring (CGM) and capillary glucose (CG) and average slope across the three tests.

Variable	Baseline (mmol/l)		Average slope (mmol/l/hour)	
	Mean	SD	Mean	SD
CGM glucose test 1	8.19 (6.70; 9.67)	2.92 (1.87; 4.63)	-4.39 (-6.27; -2.45)	4.03 (2.45; 6.66)
CGM glucose test 2	8.85 (7.09; 10.61)	4.03 (2.71; 6.19)	-3.72 (-5.82; -1.62)	5.05 (3.29; 7.86)
CGM glucose test 3	9.53 (8.14; 10.91)	2.85 (1.83; 4.53)	-4.92 (-6.75; -3.10)	4.04 (2.58; 6.43)
CG test 1	8.66 (7.19; 10.13)	2.78 (1.91; 4.13)	-5.10 (-7.81; -2.24)	6.37 (4.16; 9.89)
CG test 2	9.29 (7.28; 11.26)	4.09 (2.80; 6.14)	-5.94 (-8.93; -2.63)	7.87 (4.85; 12.92)
CG test 3	10.26 (8.99; 11.51)	2.05 (1.31; 3.25)	-7.09 (-9.45; -4.37)	4.71 (2.65; 8.05)

Data are presented as mean (95% CI).

## Data Availability

The data were obtained through employment at Nordsjællands Hospital. The data used to support the findings of this study are available from the corresponding author upon request.

## Abbreviations

CGM: Continuous glucose monitoring
CG: Capillary glucose
T1DM: Type 1 diabetes mellitus
IG: Interstitial glucose
PA: Physical activity.
